# Childhood immunization during the COVID-19 pandemic: experiences in Haiti, Lesotho, Liberia and Malawi

**DOI:** 10.2471/BLT.21.286774

**Published:** 2021-11-17

**Authors:** Emilia Connolly, Emma J Boley, Donald Luke Fejfar, Prince F Varney, Moses B Aron, Isabel R Fulcher, Wesler Lambert, Melino Ndayizigiye, Michael R Law, Jean-Claude Mugunga, Bethany Hedt-Gauthier, on behalf of the Cross-site COVID-19 Syndromic Surveillance Working Group

**Affiliations:** a Partners In Health, P.O. Box 56, Neno, Malawi.; b Partners In Health, Monrovia, Liberia.; c Partners In Health, Boston, United States of America (USA).; d Department of Global Health and Social Medicine, Harvard Medical School, Boston, USA.; e Partners In Health, Port au Prince, Haiti.; f Partners In Health, Maseru, Lesotho.; g School of Population and Public Health, University of British Columbia, Vancouver, Canada.

## Abstract

**Objective:**

To examine changes in vaccination of children younger than 1 year during the coronavirus disease 2019 (COVID-19) pandemic (March 2020–August 2021) in Haiti, Lesotho, Liberia and Malawi.

**Methods:**

We used data from health management information systems on vaccination of children aged 12 months or younger in districts supported by Partners In Health. We used data from January 2016 to February 2020 and a linear model with negative binomial distribution to estimate the expected immunization counts for March 2020–August 2021 with 95% prediction intervals, assuming no pandemic. We compared these expected levels with observed values and estimated the immunization deficits or excesses during the pandemic months.

**Findings:**

Baseline vaccination counts varied substantially by country, with Lesotho having the lowest count and Haiti the highest. We observed declines in vaccination administration early in the COVID-19 pandemic in Haiti, Lesotho and Liberia. Continued declines largely corresponded to high rates of COVID-19 infection and discrete stock-outs. By August 2021, vaccination levels had returned to close to or above expected levels in Haiti, Liberia and Lesotho; in Malawi levels remained below expected.

**Conclusion:**

Patterns of childhood immunization coverage varied by country over the course of the pandemic, with significantly lower than expected vaccination levels seen in one country during subsequent COVID-19 waves. Governments and health-care stakeholders should monitor vaccine coverage closely and consider interventions, such as community outreach, to avoid or combat the disruptions in childhood vaccination.

## Résumé

**Objectif:**

Étudier les changements en matière de vaccination des enfants âgés de moins d’un an durant la pandémie de maladie à coronavirus 2019 (COVID-19) en Haïti, au Lesotho, au Liberia et au Malawi, entre mars 2020 et août 2021.

**Méthodes:**

Nous avons utilisé les données issues des systèmes d'information pour la gestion de la santé et portant sur la vaccination des enfants de moins de 12 mois, dans les districts soutenus par l'association Partners In Health. Nous nous sommes appuyés sur des données collectées entre janvier 2016 et février 2020, ainsi que sur un modèle linéaire avec distribution binomiale négative, afin d'estimer les chiffres d'immunisation attendus pour la période allant de mars 2020 à août 2021 en appliquant des intervalles de prévision de 95%, en supposant qu'il n'y ait pas eu de pandémie. Enfin, nous avons comparé ces prévisions avec les valeurs observées, puis calculé les excédents ou déficits en la matière pendant les mois de pandémie.

**Résultats:**

Les chiffres de vaccination initiaux étaient très différents d'un pays à l'autre, le Lesotho enregistrant le plus faible taux et Haïti, le plus élevé. Nous avons constaté une diminution de l'administration des vaccins au début de la pandémie de COVID-19 en Haïti, au Lesotho et au Liberia. Les baisses continues correspondaient à des niveaux élevés d'infection à la COVID-19 et à des ruptures de stock distinctes. En août 2021, les taux de vaccination avaient repris un cours normal ou supérieur à la norme en Haïti, au Liberia et au Lesotho; au Malawi, ils sont restés inférieurs aux résultats escomptés.

**Conclusion:**

Les schémas de couverture vaccinale infantile ont varié en fonction des pays tout au long de la pandémie, avec des taux de vaccination nettement inférieurs aux prévisions dans l'un des pays durant les vagues successives de COVID-19. Les gouvernements et acteurs du secteur de la santé devraient surveiller attentivement la couverture vaccinale et envisager des interventions telles que la sensibilisation communautaire s'ils souhaitent éviter ou mettre fin aux interruptions dans la vaccination des enfants.

## Resumen

**Objetivo:**

Analizar los cambios en la vacunación de los niños menores de 1 año durante la pandemia de la coronavirosis de 2019 (COVID-19) (entre marzo de 2020 y agosto de 2021) en Haití, Lesotho, Liberia y Malawi.

**Métodos:**

Se emplearon datos de los sistemas de información de gestión sanitaria sobre la vacunación de niños de 12 meses o menos en los distritos que reciben apoyo de Partners In Health. Se usaron datos de enero de 2016 a febrero de 2020 y un modelo lineal con distribución binomial negativa para estimar las cifras de inmunización esperadas para el periodo de marzo de 2020 a agosto de 2021 con intervalos de predicción del 95 %, suponiendo que no hay pandemia. Se compararon estos niveles esperados con los valores observados y se estimaron los déficits o excesos de inmunización durante los meses de la pandemia.

**Resultados:**

El número de vacunas de referencia varió bastante según el país, siendo Lesotho el que tuvo el número más bajo y Haití el más alto. Se observó una disminución en la administración de vacunas al principio de la pandemia de la COVID-19 en Haití, Lesotho y Liberia. Las disminuciones continuas fueron en gran medida consecuencia de las altas tasas de infección por la COVID-19 y a la escasez de existencias. En agosto de 2021, los niveles de vacunación se habían acercado o superado los niveles esperados en Haití, Liberia y Lesotho; en Malawi los niveles seguían siendo inferiores a los esperados.

**Conclusión:**

Los patrones de cobertura de la inmunización infantil variaron según el país en el transcurso de la pandemia, con niveles de vacunación mucho más bajos de lo esperado en un país durante las siguientes oleadas de la COVID-19. Los gobiernos y las partes interesadas en la atención sanitaria deben supervisar de cerca la cobertura de la vacunación y contemplar intervenciones, como la divulgación en la comunidad, para evitar o superar las interrupciones de la vacunación infantil.

## ملخص

**الغرض:**

فحص التغييرات في تطعيم الأطفال الذين تقل أعمارهم عن سنة واحدة خلال جائحة مرض فيروس كورونا 2019 (كوفيد 19) (مارس/آذار 2020 إلى أغسطس/آب 2021) في هايتي وليسوتو وليبيريا وملاوي.

**الطريقة:**

قمنا باستخدام بيانات من أنظمة معلومات الإدارة الصحية بخصوص تطعيم الأطفال الذين تصل أعمارهم إلى 12 شهرًا أو أقل في المناطق التي يدعمها شركاء في الصحة. استخدمنا البيانات من يناير (كانون الثاني) 2016 إلى فبراير (شباط) 2020، ونموذج خطي بتوزيع سالب ذي الحدين لتقدير أعداد التحصين المتوقعة للفترة من مارس (آذار) 2020 إلى أغسطس (آب) 2021، مع فواصل توقع بنسبة 95%، بافتراض عدم وجود جائحة. قمنا بمقارنة هذه المستويات المتوقعة بالقيم التي تمت ملاحظتها، وقمنا بتقدير أوجه القصور أو التجاوزات في التحصين خلال أشهر الجائحة.

**النتائج:**

اختلفت أعداد التلقيح الأساسية بشكل كبير حسب الدولة، حيث سجلت ليسوتو أقل عدد، بينما سجلت هايتي الأعلى. ولاحظنا انخفاضات في إدارة التطعيم في وقت مبكر من جائحة كوفيد 19 في هايتي وليسوتو وليبيريا. وتتماشى الانخفاضات المستمرة إلى حد كبير مع المعدلات المرتفعة للإصابة بكوفيد 19، وحالات نفاد المخزون المستقلة. بحلول أغسطس (آب) 2021، عادت مستويات التطعيم إلى ما يقرب من المستويات المتوقعة أو أعلى منها في هايتي وليبيريا وليسوتو؛ بينما ظلت المستويات في ملاوي أقل من المتوقع.

**الاستنتاج:**

اختلفت أنماط تغطية تحصين الأطفال حسب الدولة خلال فترة الجائحة، مع مستويات تطعيم أقل من المتوقع بشكل ملحوظ في دولة واحدة خلال موجات كوفيد 19 التالية. يجب على الحكومات وأصحاب المصلحة في الرعاية الصحية القيام بمراقبة تغطية اللقاح عن كثب والنظر في التدخلات، مثل التوعية المجتمعية، لتجنب أو مكافحة الاضطرابات في تطعيم الأطفال.

## 摘要

**目的:**

调查海地、莱索托、利比里亚、和马拉维在新型冠状病毒肺炎 (COVID-19) 疫情期间（2020 年 3 月至 2021 年 8 月）1 岁以下的儿童疫苗接种的变化情况。

**方法:**

我们使用了健康管理信息系统提供的由健康合作伙伴支持的地区 12 个月及以下儿童接种疫苗的数据。我们使用 2016 年 1 月至 2020 年 2 月的数据和负二项分布的线性模型，来估计 2020 年 3 月至 2021 年 8 月的疫苗接种剂数，假设没有疫情，预测区间为 95%。我们将这些预期水平与观察值进行了比较，并估计了疫情期间的免疫缺陷或免疫反应过度情况。

**结果:**

不同国家的基线疫苗接种计数差别很大，莱索托的疫苗接种计数最低，海地最高。我们观察到海地、莱索托和利比里亚在新型冠状病毒肺炎疫情早期疫苗接种率下降。这种持续下降在很大程度上与新型冠状病毒肺炎感染率高和离散性缺货有关。到 2021 年 8 月，海地、莱索托和利比里亚的疫苗接种水平已恢复到接近或高于预期水平；马拉维的接种水平仍低于预期水平。

**结论:**

在疫情期间，各国的儿童免疫接种覆盖模式各不相同，在随后新型冠状病毒肺炎的反复爆发中，一个国家的疫苗接种水平明显低于预期水平。政府和医疗卫生利益相关者应密切监测疫苗接种覆盖率，并考虑采取干预措施，如社区外联，以避免或应对儿童疫苗接种中断的问题。

## Резюме

**Цель:**

Изучить изменения в вакцинации детей младше 1 года во время пандемии коронавирусной инфекции 2019 года (COVID-19) (март 2020 г. — август 2021 г.) в Гаити, Лесото, Либерии и Малави.

**Методы:**

Авторы использовали данные из информационных систем управления в области здравоохранения о вакцинации детей в возрасте 12 месяцев и младше в районах, которые получают поддержку от организации «Партнеры во имя здоровья». Авторы использовали данные с января 2016 г. по февраль 2020 г., а также линейную модель с отрицательным биномиальным распределением для оценки ожидаемого числа иммунизаций на март 2020 г. — август 2021 г. с 95%-ми интервалами прогнозирования при условии отсутствия пандемии. Авторы сравнили эти ожидаемые уровни с наблюдаемыми значениями и оценили дефицит или избыток иммунизации в месяцы пандемии.

**Результаты:**

Исходные показатели вакцинации существенно различались в разных странах: самый низкий показатель был в Лесото, а самый высокий — в Гаити. В начале пандемии COVID-19 наблюдалось снижение уровня вакцинации в Гаити, Лесото и Либерии. Продолжающееся снижение в значительной степени соответствовало высоким показателям заражения COVID-19 и дискретным дефицитам. К августу 2021 г. в Гаити, Либерии и Лесото уровни вакцинации вернулись к ожидаемым уровням или превысили их. В Малави уровни остались ниже ожидаемых.

**Вывод:**

На протяжении пандемии характер охвата детей иммунизацией отличался в разных странах, при этом в одной стране во время последующих волн COVID-19 уровень вакцинации был значительно ниже, чем ожидалось. Правительствам и заинтересованным сторонам в сфере здравоохранения следует внимательно следить за охватом вакцинацией и предусматривать информационно-просветительскую работу с населением во избежание сбоев в вакцинации детей или для противостояния таким сбоям.

## Introduction

The coronavirus disease 2019 (COVID-19) pandemic has severely affected health services globally, leading to concerns about disruptions to essential services such as immunizations.^1^ Vaccinations, particularly those given in the first year of life, result in substantial reductions in mortality and are among the most cost-effective health interventions in low- and middle-income countries.^2^^–^^4^ Before the pandemic, inequity in full vaccination coverage in low- and middle-income countries persisted due to inadequate health infrastructure, insufficient human resources and supply chain disruptions. People living in the poorest households and in remote areas are least likely to have optimal vaccination coverage and uptake.^5^ These inequities were exacerbated in previous health emergencies, such as the H1N1 influenza and Ebola virus disease epidemics,^6^^,^^7^ and vaccine interruptions have led to secondary disease outbreaks.^8^^,^^9^ Early COVID-19 pandemic models predicted immunization interruptions and raised alarms about the possibility of increased mortality as a result.^9^^–^^12^ For example, one scenario predicted that for every one excess COVID-19 death acquired during visits for routine vaccination, 84 deaths could be prevented by sustaining routine childhood immunization in Africa.^10^

Governments have adopted policies such as curfews, travel bans and school closures to mitigate the spread of severe acute respiratory syndrome coronavirus 2 (SARS-CoV-2).^13^^,^^14^ However, these interventions can have unintended effects on health-service delivery. For example, of 105 countries included in the World Health Organization (WHO) pulse survey in mid-2020, 50% reported partial disruptions and 10% reported severe disruptions in facility-based immunizations.^15^ Some early studies from both low- and middle-income countries and high-income countries observed reductions in vaccinations depending on geography and the corresponding pandemic prevention measures reported.^16^^–^^22^ Of these studies, we identified just two studies showing rebounds in coverage alongside catch-up campaigns and the lifting of social distancing measures.^19^^,^^20^ Important gaps in the literature exist, including limited sustained monitoring beyond initial prevention measures, no on-the-ground investigations in low- and middle-income countries outside Asia and little investigation within specific populations at risk.^23^

Recognizing the potential disruption of childhood vaccinations due to COVID-19, we longitudinally monitored vaccination administration in four countries with geographic and population differences: Haiti, Lesotho, Liberia and Malawi. Within these countries, our teams support health-care delivery in specific districts, primarily sites that are rural and hard to reach – the communities usually most at risk of disruption of services during an emergency. In this study, we examined how vaccinations of children younger than 1 year were affected during the COVID-19 pandemic and discuss potential ways immunization programmes can maintain essential services during acute health crises.

## Methods

### Study sites

Partners In Health is a global nongovernmental organization working through a so-called accompaniment model, employing close partnership with national and local governments to build strong and equitable health systems for the most vulnerable communities.^24^ Partners In Health does not operate health facilities, but integrates into the public facility operations of health ministries. Therefore, support in the four countries included in this study: Haiti, Lesotho, Liberia and Malawi – which served 10 districts, 41 facilities and about 6.4 million people, is adapted by country to fit the needs of the country’s health system. Here we focus on districts supported by Partners In Health because (i) we use the health-service utilization assessment methods presented in this study to routinely monitor the pandemic there and therefore can rapidly obtain data and results; and (ii) as we are integrated into programming, we can assess the effect of the COVID-19 pandemic in the context of these locales. While Partners In Health operates in many countries, we focused on these four countries due to the availability of their data, their capacity to provide data and their prioritization of childhood immunization.

The study was approved by: Zanmi Lasante Institutional Review Board (ZLIRB, protocol number ZLIRB01252021 – Retrospective studies related to COVID-19 in Partners In Health/Zanmi Lasante-supported regions of Haiti); the Ministry of Health Research and Ethics Committee of Lesotho (protocol number 103–2020 – Retrospective cohort studies related to COVID-19 in Partners In Health); University of Liberia-Pacific Institute for Research & Evaluation Institutional Review Board (UL-PIRE IRB, protocol number 17–06–048 – Evaluation of PIH-supported clinical delivery in Liberia); and by the National Health Science Research Committee of Malawi (protocol number 1216 – Evaluation of clinical care in Neno District-Malawi).

### Analytical approach

As part of our support for the response to the COVID-19 pandemic at these facilities, we developed methods for ongoing monitoring of indicators of health-service utilization captured in existing routine health information systems.^25^^–^^27^

Full details of our analytical approach are given elsewhere^25^ and further specifications for this study are detailed in the supplementary material in the data repository.^28^ In brief, we modelled monthly immunization counts for each facility with a negative binomial regression accounting for yearly trends and seasonality using historical data from January 2016 to February 2020, except for Haiti which started from January 2017. We did not include terms for autocorrelation, as no autocorrelation was detected in the residuals of these models. We used these models to extrapolate immunization counts to 2020 and 2021, providing estimates with 95% prediction intervals of what counts we would expect in the absence of the pandemic, aggregated across the included sites at the country level. We also computed the cumulative difference in number of vaccinations (observed – predicted vaccination counts) and per cent difference in vaccinations during the following periods of the COVID-19 pandemic: early (March–August 2020), middle (September 2020–February 2021), late (March–August 2021) and total (March 2020–August 2021). An observation was flagged as a statistically significant deviation from expected if it was less than zero and the 95% prediction interval did not contain zero.

We excluded a facility for a specific vaccine dose if: (i) that vaccine dose was missing data for more than 20% of baseline months; or (ii) that vaccine dose was missing data for any of the months of the evaluation period (March 2020–August 2021). For indicators missing baseline data, we fitted models assuming the data were missing completely at random.^25^ Vaccine-dose data excluded due to missing data at the facility level were also excluded from the country-level summary reported in this study. Of the 301 facility-indicator combinations, eight (2.7%) were excluded. Data were checked for outliers and reviewed by site staff. All analyses and visualizations were done in R v4.0.4 (R Foundation, Vienna, Austria).

### Immunization vaccine-dose combinations

We considered 14 vaccine-dose combinations administered to children younger than 1 year in the four countries (Table 1 available at: https://www.who.int/publications/journals/bulletin/). The combinations included bacillus Calmette–Guerin (BCG) vaccine, polio vaccine (oral polio vaccine or inactivated polio vaccine; doses 0–3), pentavalent vaccine (hepatitis B–*Haemophilus influenzae* type B–diphtheria–tetanus–pertussis; doses 1–3), pneumococcal vaccine (doses 1–3), rotavirus vaccine (doses 1 and 2) and measles vaccine (dose 1). We report results for each vaccine dose by country; some combinations are not included for specific countries, as indicated in Table 2. We grouped vaccines into five classes based on age at administration: at birth, at 6 weeks, at 10 weeks, at 14 weeks and at 36 weeks.

**Table 1:** National- and district-level features of countries included in the study on child immunization during the COVID-19 pandemic, 2020–2021

Feature	Haiti	Lesotho	Liberia	Malawi
**Partners In Health**				
Function at district level	Assists government to provide clinical and community care in 15 clinics and hospitals; supports a teaching hospital (University of Mirebalais)	Supports government-led clinical and community care with emphasis on HIV, multidrug-resistant tuberculosis and health systems strengthening	Accompanies the government in the hardest-to-reach areas through the continuum of care	Accompanies the government across the whole health system in the most remote and underserved district in the country
Supported districts included in study	Central plateau and lower Artibonite	Mohale’s Hoek, Thaba Tseka, Mokhotlong, and Qacha’s Nek	Harper, Pleebo, and Karluway 1	Neno
**National COVID-19 situation**				
First COVID case	March 2020	May 2020	March 2020	April 2020
Restriction period(s)	March–May 2020	March–May 2020; January–February 2021	April–July 2020	Proposed April 2020 but never enacted
Ongoing prevention measures	Stay at home and curfews recommended with infection waves; limits on numbers in gatherings	Limits on numbers in gatherings and on transport; curfews; screening in public places	Limits on numbers in gatherings; restrictions on intercounty movement; curfews; screening in public places	Limits on numbers in gatherings and public transport; curfews with infection waves
COVID-19 stringency index out of 100,^29^ range (month)	93.50 (April 2020) to 21.30 (August 2020)	90.74 (April 2020) to 28.70 (May 2021)	87.96 (April 2020) to 35.19 (January 2021)	64.81 (April 2020) to 31.48 (May 2021)
Cumulative COVID-19 cases,^30^^,^^31^ no. per 1 000 000 people^a^	1806.50	6667.23	1079.88	3073.44
Cumulative case fatality rate,^30^^,^^31^ % (total deaths/cumulative cases)^a^	2.80 (586/20 896)	2.80 (403/14 395)	4.38 (245/5594)	3.60 (2177/60 494)
Infection waves,^30^^,^^31^ no. new confirmed cases rolling 7-day peak per 1 000 000 people (month year)	16.59 (June 2020); 7.18 (January 2021); 16.46 (June 2021)	18.73 (August 2020); 180.70 (January 2021); 49.76 (July 2021)	4.28 (June 2020); 31.00 (July 2021)	5.60 (July 2020); 50.53 (January 2021); 37.75 (June 2021)
**District situation and response**				
Prevention measures in supported districts, none, some, all	None to some	None to some	Some	None to some
Vaccines with reported stock-outs	BCG (November 2020–February 2021)	BCG and polio vaccines (October 2020)	Pentavalent (December 2020–January 2021)	None
District strategies to increase vaccination uptake	Consistent community outreach even with waves of infection; community sensitization and mobilization; COVID-19 screening at facility entrance and clinical care protocols	Consistent community outreach even with waves of infection; integrated primary health-care services including immunization; screening at facility entrance	Consistent community outreach even with waves of infection; community sensitization; catch-up campaign with motivational food package and transport support	Community outreach only with low levels of infection; community sensitization and mobilization; screening at facility entrance and infection control; transport and logistical support
Specific catch-up campaigns in study period	No district or country-wide campaigns	Reinforced community outreach November 2020; no country-wide campaigns	District catch-up campaign December 2020; no coordinated country-wide campaigns	No district or country-wide campaigns

BCG: bacillus Calmette–Guerin; COVID-19: coronavirus disease 2019; HIV: human immunodeficiency virus.

^a^ As of 31 August 2021.

**Table 2:** National vaccination schedules for children younger than 12 months of age, by country

Vaccine (dose)	Age to receive vaccine, weeks			
	Haiti	Lesotho	Liberia	Malawi
Bacillus Calmette–Guerin (1)	0	0	0	0
Oral or inactivated polio (0)	0	0	0	0^a^
Oral or inactivated polio (1)	6	6	6	6
Oral or inactivated polio (2)	10	10^a^	10	10
Oral or inactivated polio (3)	14	14	14	14
Pentavalent (1)	6	6	6	6
Pentavalent (2)	10	10^a^	10	10
Pentavalent (3)	14	14	14	14
Pneumococcal conjugate (1)	6	6^a^	6	6
Pneumococcal conjugate (2)	10	10^a^	10	10
Pneumococcal conjugate (3)	14	14^a^	14	14
Rotavirus (1)	6	6^a^	6	6
Rotavirus (2)	10	10^a^	10	10
Measles (1)	36	36	36	36

^a^ No immunization data available.

## Results

### Vaccination levels before COVID-19

Table 3 reports the monthly volume of vaccinations administered during the baseline period (January 2016 to February 2020). The volume varied substantially by country due to catchment size, with Lesotho having the lowest number of monthly vaccinations, from 63.5 (interquartile range, IQR: 51.5 to 71.0) oral polio or inactivated polio vaccines (dose 0) administered a month to 100.0 (IQR: 79.0 to 106.0) pentavalent vaccine (dose 1) administered a month. Haiti had the highest number of vaccines administered, from 289.7 (IQR: 143.1 to 464.5) pneumococcal vaccines (dose 3) administered a month to 947.0 (IQR: 797.0 to 1114.5) BCG vaccines administered a month.

**Table 3:** Vaccinations administered monthly by country before the COVID-19 pandemic, January 2016–February 2020

Vaccine (dose)	Vaccinations administered a month, median no. (IQR)			
	Haiti	Lesotho	Liberia	Malawi
Bacillus Calmette–Guerin (1)	947.0 (797.0 to 1 114.5)	79.5 (66.2 to 93.8)	193.0 (158.2 to 233.8)	488.5 (422.8 to 549.8)
Oral or inactivated polio (0)	338.5 (245.8 to 411.0)	63.5 (51.5 to 71.0)	156.5 (125.0 to 186.5)	NA
Oral or inactivated polio (1)	766.5 (609.5 to 1 045.0)	95.0 (84.2 to 106.8)	218.0 (175.5 to 247.8)	501.5 (434.5 to 743.0)
Oral or inactivated polio (2)	707.5 (453.5 to 900.5)	NA	203.0 (160.8 to 229.0)	435.5 (361.8 to 467.8)
Oral or inactivated polio (3)	491.5 (352.2 to 590.5)	89.5 (76.0 to 95.8)	208.5 (174.5 to 243.0)	463.5 (394.2 to 501.2)
Pentavalent (1)	791.5 (654.2 to 1 071.0)	100.0 (79.0 to 106.0)	218.0 (175.5 to 247.8)	474.0 (388.2 to 514.0)
Pentavalent (2)	700.0 (538.8 to 909.2)	NA	203.0 (160.8 to 230.5)	438.0 (367.2 to 463.8)
Pentavalent (3)	612.0 (532.8 to 835.8)	86.5 (74.5 to 95.8)	208.5 (175.2 to 243.0)	462.5 (397.2 to 506.0)
Pneumococcal conjugate (1)	635.7 (381.1 to 857.0)	NA	218.0 (175.5 to 246.2)	466.5 (401.2 to 519.0)
Pneumococcal conjugate (2)	443.3 (208.0 to 668.8)	NA	203.0 (160.8 to 229.0)	440.5 (366.5 to 463.0)
Pneumococcal conjugate (3)	289.7 (143.1 to 464.5)	NA	208.5 (174.5 to 243.0)	459.5 (393.5 to 507.0)
Rotavirus (1)	743.5 (592.2 to 986.5)	NA	216.5 (159.2 to 242.2)	470.5 (368.2 to 503.2)
Rotavirus (2)	675.0 (484.0 to 843.5)	NA	197.5 (129.2 to 223.5)	431.5 (354.0 to 471.8)
Measles (1)	564.0 (489.0 to 690.0)	78.0 (62.2 to 92.8)	181.5 (139.0 to 222.2)	259.5 (0.0 to 486.8)

Covid-19: coronavirus disease 2019; IQR: interquartile range; NA: data not available.

### Vaccinations, March–August 2020

**Fig. 1:**
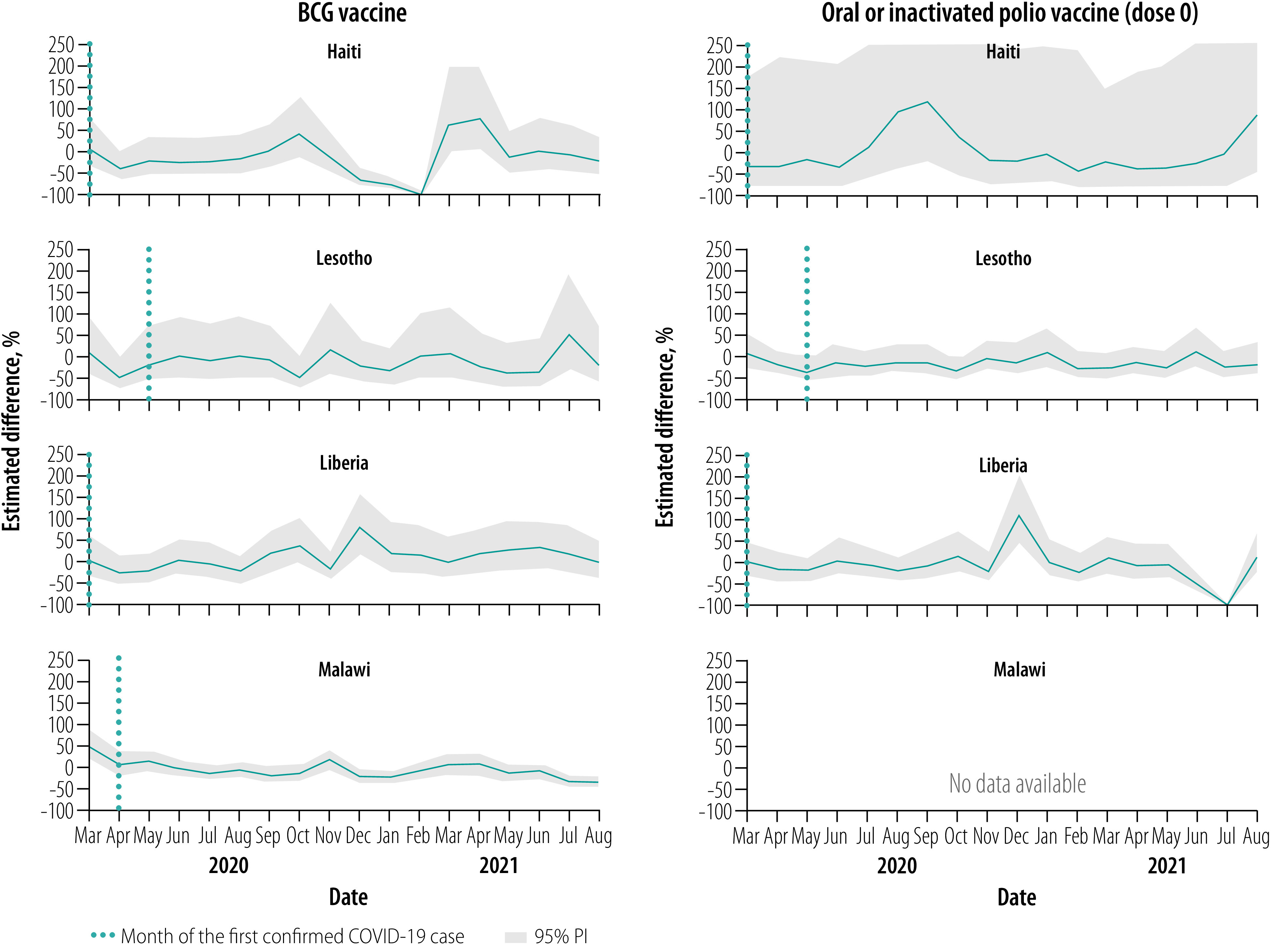
Estimated per cent difference from expected in vaccine doses given at age 0 weeks, by month and country, March 2020–August 2021

BCG: Bacillus Calmette–Guerin; COVID-19: coronavirus disease 2019; PI: prediction interval.

Note: We only show 95% prediction intervals within 250% difference (details in data repository).^28^

**Fig. 2:**
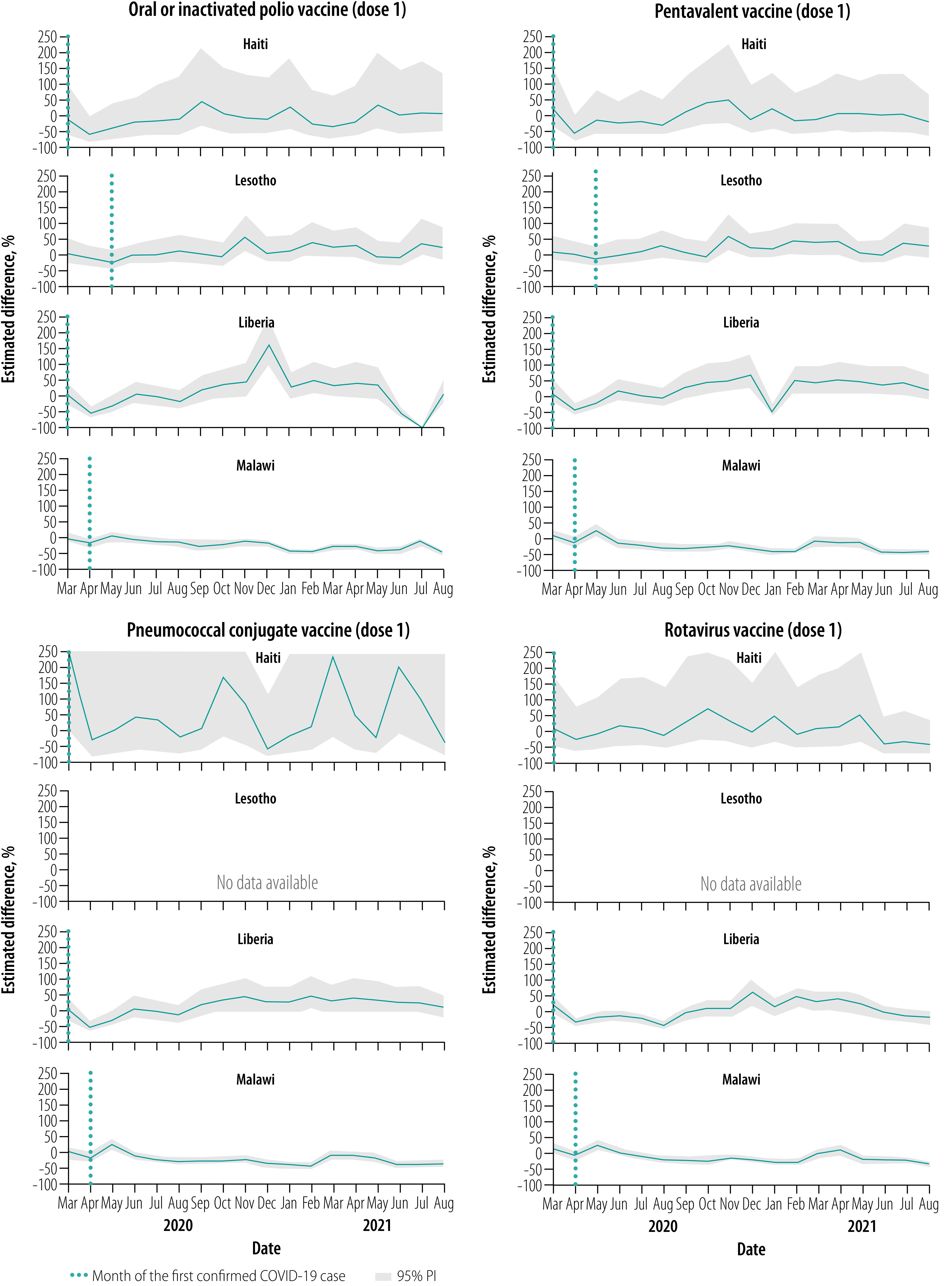
Estimated per cent difference from expected in vaccine doses given at 6 weeks, by month and country, March 2020–August 2021

COVID-19: coronavirus disease 2019; PI: prediction interval.

Note: We only show 95% prediction intervals within 250% difference (details in data repository).^28^

**Fig. 3:**
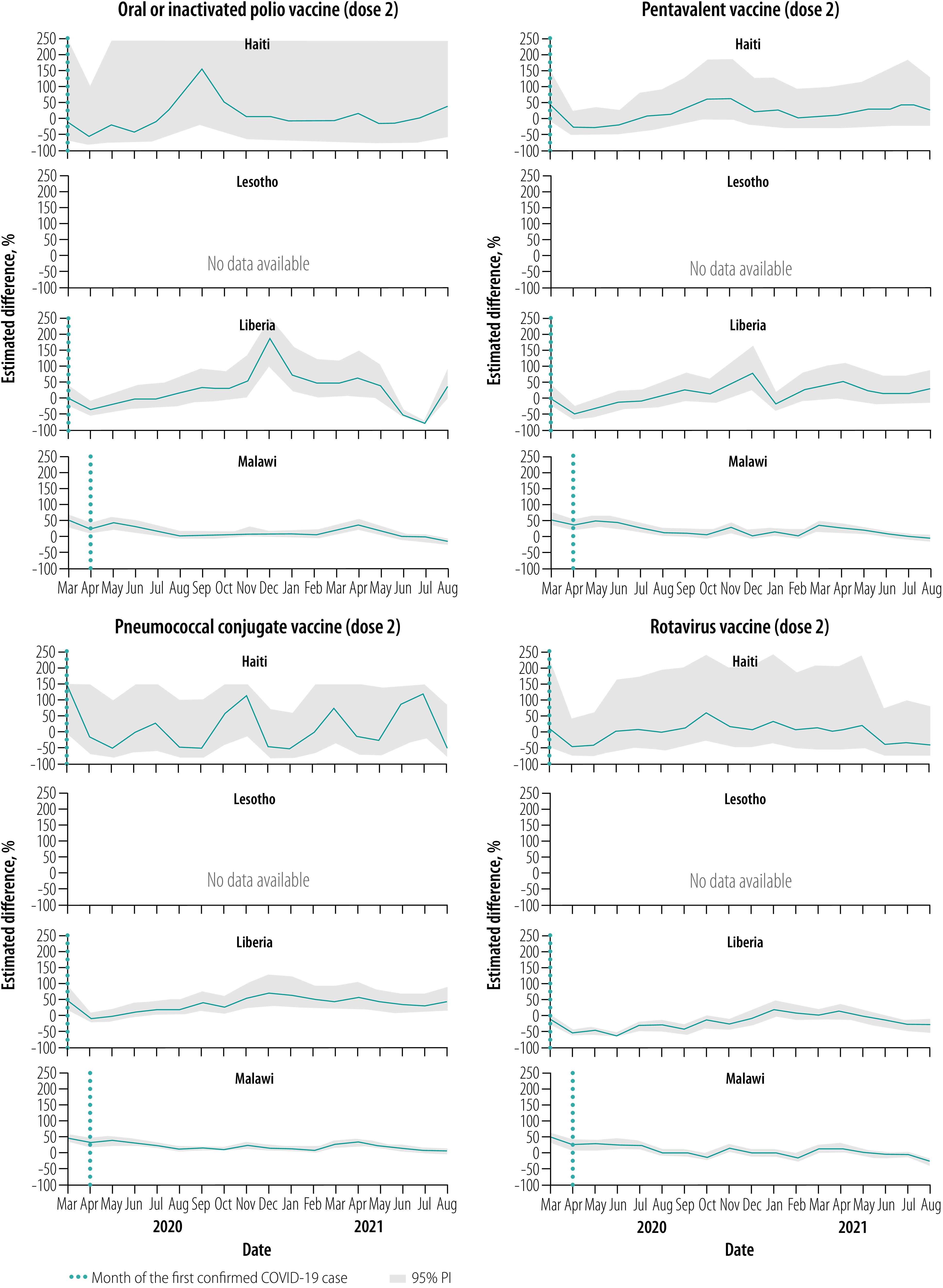
Estimated per cent difference from expected in vaccine doses given at 10 weeks, by month and country, March 2020–August 2021

COVID-19: coronavirus disease 2019; PI: prediction interval.

Note: We only show 95% prediction intervals within 250% difference (details in data repository).^28^

**Fig. 4:**
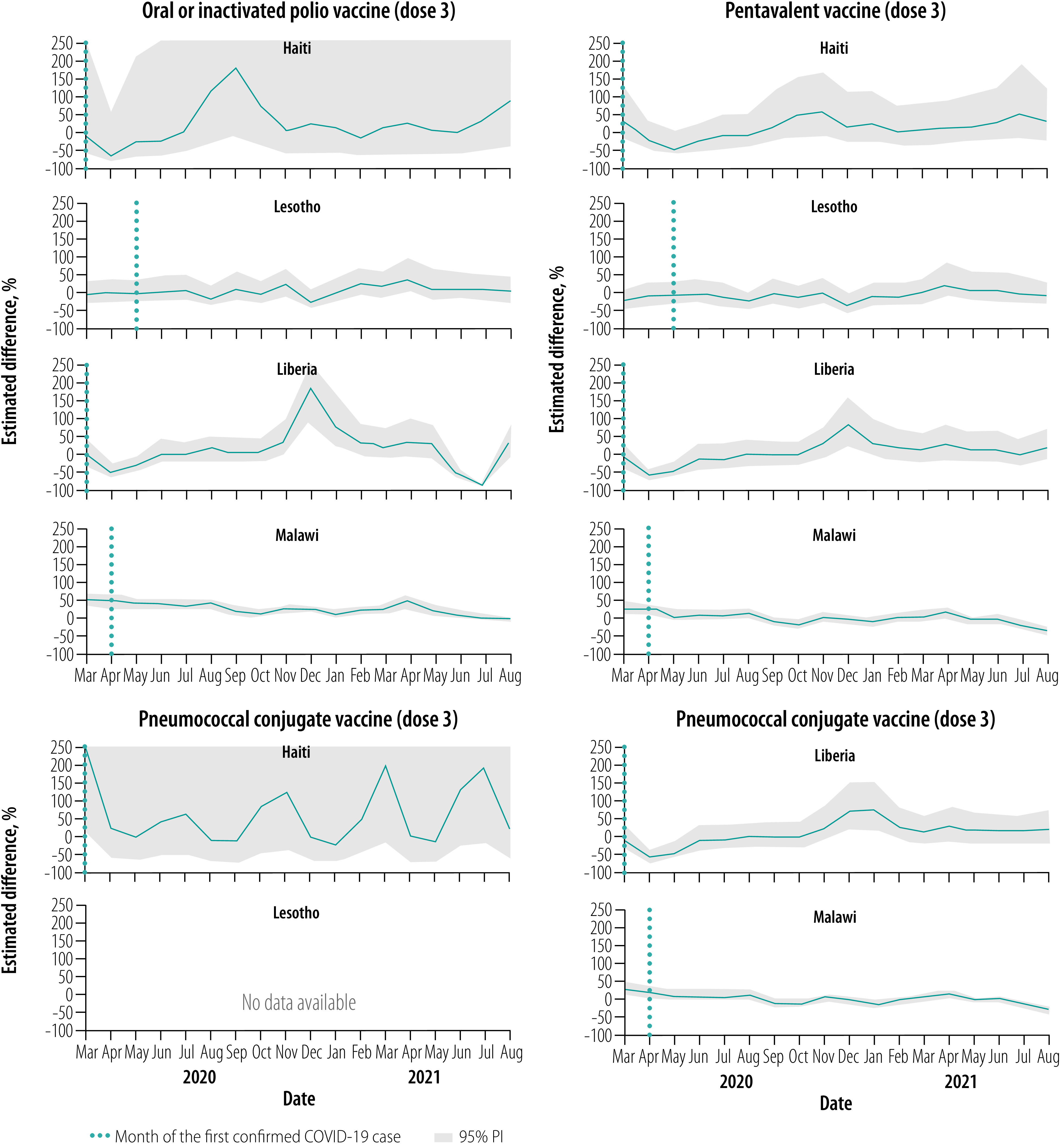
Estimated per cent difference from expected in vaccine doses given at 14 weeks, by month and country, March 2020–August 2021

COVID-19: coronavirus disease 2019; PI: prediction interval.

Notes: We only show 95% prediction intervals within 250% difference (details in data repository).^28^

**Fig. 5:**
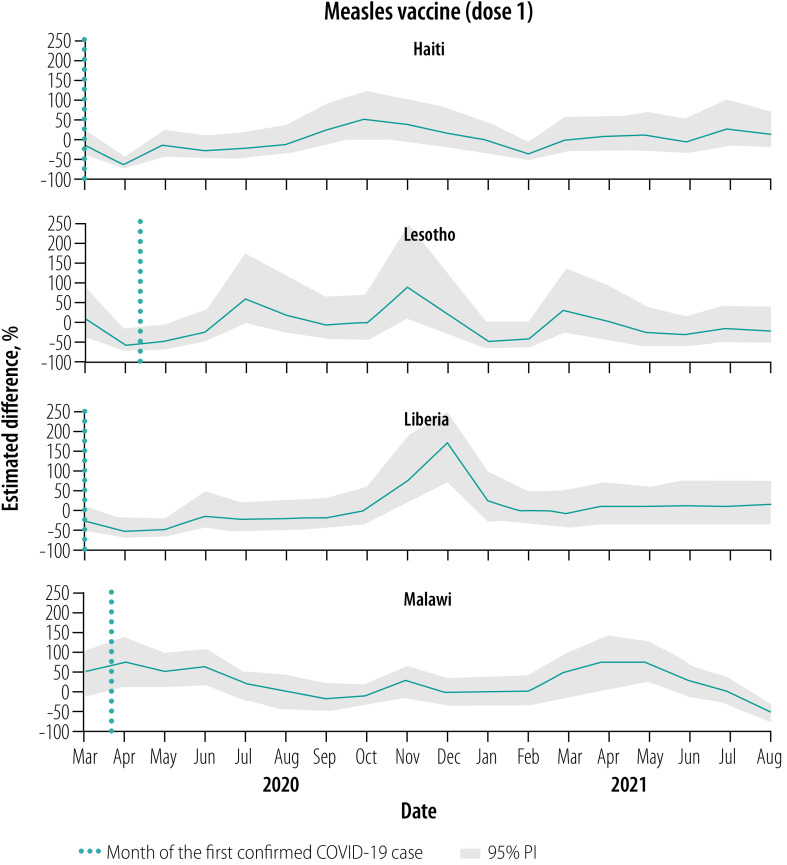
Estimated per cent difference from expected in vaccine doses given at 36 weeks, by month and country, March 2020–August 2021

COVID-19: coronavirus disease 2019; PI: prediction interval.

Overall, for all vaccines, March 2020 levels were close to expected but were followed by notable declines relative to expected levels throughout this early evaluation period, apart from in Malawi (Fig. 1, Fig. 2, Fig. 3, Fig. 4 and Fig. 5). No country site had a statistically significant early decline in vaccines administered at birth (Fig. 1). However, Haiti, Lesotho and Liberia had a statistically significant decline in measles vaccinations early in this period with an upward trend at the end of the period (Fig. 5). For March–August 2020, the median (range) cumulative percentage difference in the 14 vaccine-dose combinations reported was 17.4% (−35.5% to 18.3%) less than expected for Haiti, 7.0% (−16.6% to −3.4%) less for Lesotho, 17.0% (−39.1% to −8.0%) less for Liberia and 13.7% (2.4% to 33.6%) greater for Malawi (Table 4; available at: https://www.who.int/publications/journals/bulletin/; details in data repository).^28^

**Table 4:** Cumulative difference in vaccinations from the baseline period (January 2016–February 2020) in countries, 2020–2021

Country and vaccine (dose)	March–August 2020				September 2020–February 2021				March–August 2021				March 2020–August 2021		
	Cumulative observed counts, true value	Cumulative difference, estimated counts (95% PI)	Difference over expected, estimated % (95% PI)		Cumulative observed counts, true value	Cumulative difference, estimated counts (95% PI)	Difference over expected, estimated % (95% PI)		Cumulative observed counts, true value	Cumulative difference, estimated counts (95% PI)	Difference over expected, estimated % (95% PI)		Cumulative observed counts, true value	Cumulative difference, estimated counts (95% PI)	Difference over expected, estimated % (95% PI)
**Haiti**															
Bacillus Calmette–Guerin (1)	4904	-1083.5 (-2782.2 to 165)	-18.1% (-36.2 to 3.5)		4020	-2414 (-4419 to -901.6)	-37.5% (-52.4 to -18.3)		7142	939 (-1235.8 to 2452.1)	15.1% (-14.7 to 52.3)		16066	-2666 (-7471.5 to 963.1)	-14.2% (-31.7 to 6.4)
Oral or inactivated polio (0)	1608	-337.5 (-1996.1 to 524.8)	-17.3% (-55.4 to 48.4)		2103	-159.5 (-2160.1 to 898.8)	-7% (-50.7 to 74.6)		1567	-429 (-2923.7 to 516.1)	-21.5% (-65.1 to 49.1)		5278	-953.5 (-5926.8 to 1482)	-15.3% (-52.9 to 39)
Oral or inactivated polio (1)	7615	-2301.5 (-6452.3 to 746.6)	-23.2% (-45.9 to 10.9)		11390	587.5 (-4841.4 to 3799.5)	5.4% (-29.8 to 50.1)		10691	-32.5 (-6786.4 to 3704.1)	-0.3% (-38.8 to 53)		29696	-2425 (-14999 to 6431.5)	-7.5% (-33.6 to 27.6)
Oral or inactivated polio (2)	3315	-1825.5 (-6535.2 to 879.1)	-35.5% (-66.3 to 36.1)		5057	-166.5 (-5813.9 to 2609.6)	-3.2% (-53.5 to 106.6)		5054	-801.5 (-9684.3 to 2483.3)	-13.7% (-65.7 to 96.6)		13426	-2714 (-18131.2 to 4662.7)	-16.8% (-57.5 to 53.2)
Oral or inactivated polio (3)	2313	-982 (-4051.5 to 688.3)	-29.8% (-63.7 to 42.4)		3499	211 (-3509.4 to 1881.6)	6.4% (-50.1 to 116.3)		3650	-74 (-4643.6 to 1967.5)	-2% (-56 to 116.9)		9462	-1186 (-9607.1 to 3615.6)	-11.1% (-50.4 to 61.8)
Pentavalent (1)	4934	-952 (-3036.5 to 697.5)	-16.2% (-38.1 to 16.5)		7212	898 (-2021 to 2650.2)	14.2% (-21.9 to 58.1)		6365	-30.5 (-3180.3 to 2004.8)	-0.5% (-33.3 to 46)		18511	-248.5 (-6455.2 to 4600.5)	-1.3% (-25.9 to 33.1)
Pentavalent (2)	4384	-856 (-2568.1 to 484.2)	-16.3% (-36.9 to 12.4)		5482	820 (-1132.2 to 2083.3)	17.6% (-17.1 to 61.3)		5947	442.5 (-1988.5 to 2073.7)	8% (-25.1 to 53.5)		15813	252.5 (-4170.2 to 3995.4)	1.6% (-20.9 to 33.8)
Pentavalent (3)	3722	-993.5 (-2485.8 to 107.3)	-21.1% (-40 to 3)		4375	596.5 (-652.8 to 1524.4)	15.8% (-13 to 53.5)		5522	647.5 (-1378.7 to 1959)	13.3% (-20 to 55)		13619	130.5 (-3519.5 to 3002.8)	1% (-20.5 to 28.3)
Pneumococcal conjugate (1)	5313	371 (-4401.6 to 2858.1)	7.5% (-45.3 to 116.4)		4801	-195.5 (-4019 to 1965.7)	-3.9% (-45.6 to 69.3)		6773	1924.5 (-2280.7 to 4320)	39.7% (-25.2 to 176.2)		16887	1694.5 (-6162 to 6835.5)	11.2% (-26.7 to 68)
Pneumococcal conjugate (2)	4385	677.5 (-1952.2 to 2148.1)	18.3% (-30.8 to 96)		3690	77 (-2424.3 to 1555)	2.1% (-39.6 to 72.8)		5213	1469.5 (-1111 to 2896.3)	39.3% (-17.6 to 125)		13288	1986.5 (-2885.4 to 5166.3)	17.6% (-17.8 to 63.6)
Pneumococcal conjugate (3)	3496	533 (-2077.2 to 1997.2)	18% (-37.3 to 133.3)		2900	200.5 (-2265.3 to 1371.9)	7.4% (-43.9 to 89.8)		4148	1251 (-1388.3 to 2688.1)	43.2% (-25.1 to 184.1)		10544	1749.5 (-2726.8 to 4706.6)	19.9% (-20.5 to 80.6)
Rotavirus (1)	4621	-737 (-3478.8 to 1232.1)	-13.8% (-42.9 to 36.4)		6297	733 (-2560 to 2645.6)	13.2% (-28.9 to 72.5)		4859	-863.5 (-5157.4 to 1434.3)	-15.1% (-51.5 to 41.9)		15777	-1003 (-9018.9 to 4151.9)	-6% (-36.4 to 35.7)
Rotavirus (2)	3775	-798.5 (-3486.7 to 977.5)	-17.5% (-48 to 34.9)		4607	640.5 (-1637.6 to 2085.5)	16.1% (-26.2 to 82.7)		4114	-635.5 (-4372.7 to 1401.6)	-13.4% (-51.5 to 51.7)		12496	-1114 (-7852.5 to 3672.1)	-8.2% (-38.6 to 41.6)
Measles (1)	2700	-1004.5 (-1855.1 to -377.4)	-27.1% (-40.7 to -12.3)		4181	428 (-426.6 to 1130.3)	11.4% (-9.3 to 37.1)		4022	175 (-918.6 to 961.1)	4.5% (-18.6 to 31.4)		10903	-478 (-2704 to 1286.5)	-4.2% (-19.9 to 13.4)
**Lesotho**															
Bacillus Calmette–Guerin (1)	417	-51 (-211.6 to 70)	-10.9% (-33.7 to 20.2)		429	-80 (-250 to 48.5)	-15.7% (-36.8 to 12.8)		426	-50 (-271.6 to 88)	-10.5% (-38.9 to 26)		1272	-195 (-598.2 to 154.5)	-13.3% (-32 to 13.8)
Oral or inactivated polio (0)	339	-67.5 (-146.5 to -1)	-16.6% (-30.2 to -0.3)		384	-71.5 (-162 to 4.6)	-15.7% (-29.7 to 1.2)		360	-73 (-171.1 to 4.5)	-16.9% (-32.2 to 1.3)		1083	-211.5 (-427.5 to -40.5)	-16.3% (-28.3 to -3.6)
Oral or inactivated polio (1)	457	-16 (-93.5 to 59)	-3.4% (-17 to 14.8)		604	89.5 (-5.5 to 175)	17.4% (-0.9 to 40.8)		520	72 (-13.5 to 155)	16.1% (-2.5 to 42.5)		1581	145.5 (-43 to 330)	10.1% (-2.7 to 26.4)
Oral or inactivated polio (2)	NA	NA	NA		NA	NA	NA		NA	NA	NA		NA	NA	NA
Oral or inactivated polio (3)	475	-26 (-107.5 to 51)	-5.2% (-18.5 to 12)		505	7 (-79.5 to 81)	1.4% (-13.6 to 19.1)		554	66 (-30.1 to 138)	13.5% (-5.2 to 33.2)		1534	45 (-170.7 to 212.6)	3% (-10 to 16.1)
Pentavalent (1)	461	-26 (-115.5 to 48)	-5.3% (-20 to 11.6)		604	79 (-25 to 168)	15% (-4 to 38.5)		520	58 (-39.6 to 147)	12.6% (-7.1 to 39.4)		1585	106.5 (-109 to 310.6)	7.2% (-6.4 to 24.4)
Pentavalent (2)	NA	NA	NA		NA	NA	NA		NA	NA	NA		NA	NA	NA
Pentavalent (3)	422	-55 (-130.5 to 19.5)	-11.5% (-23.6 to 4.9)		430	-56 (-137.5 to 22)	-11.5% (-24.2 to 5.4)		495	26.5 (-71.5 to 104.6)	5.7% (-12.6 to 26.8)		1347	-84 (-280.5 to 91.1)	-5.9% (-17.2 to 7.3)
Pneumococcal conjugate (1)	NA	NA	NA		NA	NA	NA		NA	NA	NA		NA	NA	NA
Pneumococcal conjugate (2)	NA	NA	NA		NA	NA	NA		NA	NA	NA		NA	NA	NA
Pneumococcal conjugate (3)	NA	NA	NA		NA	NA	NA		NA	NA	NA		NA	NA	NA
Rotavirus (1)	NA	NA	NA		NA	NA	NA		NA	NA	NA		NA	NA	NA
Rotavirus (2)	NA	NA	NA		NA	NA	NA		NA	NA	NA		NA	NA	NA
Measles (1)	453	-34 (-161.1 to 64)	-7% (-26.2 to 16.5)		361	-50 (-184.5 to 55.5)	-12.2% (-33.8 to 18.2)		416	-78 (-250 to 52)	-15.8% (-37.5 to 14.3)		1230	-164 (-495 to 100.5)	-11.8% (-28.7 to 8.9)
**Liberia**															
Bacillus Calmette–Guerin (1)	1123	-97 (-365 to 108.5)	-8% (-24.5 to 10.7)		1129	247.5 (70.5 to 407.5)	28.1% (6.7 to 56.5)		1407	242 (-73 to 463.5)	20.8% (-4.9 to 49.1)		3659	398 (-211.3 to 869.9)	12.2% (-5.5 to 31.2)
Oral or inactivated polio (0)	927	-89 (-276.5 to 63.5)	-8.8% (-23 to 7.4)		832	102.5 (-37 to 218.6)	14.1% (-4.3 to 35.6)		785	-206 (-418.6 to -32)	-20.8% (-34.8 to -3.9)		2544	-197.5 (-673.5 to 161.1)	-7.2% (-20.9 to 6.8)
Oral or inactivated polio (1)	1198	-171 (-400.6 to 45.5)	-12.5% (-25.1 to 4)		1774	630.5 (438.5 to 806)	55.1% (32.8 to 83.3)		1292	-50.5 (-354.7 to 179.2)	-3.8% (-21.5 to 16.1)		4264	399 (-138 to 897.5)	10.3% (-3.1 to 26.7)
Oral or inactivated polio (2)	1062	-212 (-568.1 to 3)	-16.6% (-34.9 to 0.3)		1707	616.5 (355.5 to 837.5)	56.5% (26.3 to 96.3)		1232	-53 (-412.1 to 211.6)	-4.1% (-25.1 to 20.7)		4001	328 (-327.8 to 874)	8.9% (-7.6 to 28)
Oral or inactivated polio (3)	1041	-292 (-571.1 to -89.4)	-21.9% (-35.4 to -7.9)		1717	517.5 (272.4 to 720.1)	43.1% (18.9 to 72.2)		1167	-184 (-533.3 to 47.1)	-13.6% (-31.4 to 4.2)		3925	23.5 (-585.7 to 525.2)	0.6% (-13 to 15.4)
Pentavalent (1)	1190	-169.5 (-406.6 to 11.6)	-12.5% (-25.5 to 1)		1423	274 (84.9 to 450.1)	23.8% (6.3 to 46.3)		1768	429 (161.8 to 666.1)	32% (10.1 to 60.5)		4381	529 (11.7 to 979.1)	13.7% (0.3 to 28.8)
Pentavalent (2)	1063	-223.5 (-487.8 to -27)	-17.4% (-31.5 to -2.5)		1398	301 (88.4 to 488)	27.4% (6.8 to 53.6)		1655	372.5 (62.9 to 594.6)	29% (4 to 56.1)		4116	436.5 (-145.8 to 882.9)	11.9% (-3.4 to 27.3)
Pentavalent (3)	1035	-312.5 (-594.5 to -105)	-23.2% (-36.5 to -9.2)		1492	276 (8 to 459)	22.7% (0.5 to 44.4)		1573	195 (-91.1 to 430.5)	14.2% (-5.5 to 37.7)		4100	139.5 (-479.5 to 664.5)	3.5% (-10.5 to 19.3)
Pneumococcal conjugate (1)	1196	-174 (-405.5 to 0)	-12.7% (-25.3 to 0)		1533	384 (197.4 to 558.1)	33.4% (14.8 to 57.3)		1769	417 (129.4 to 642.5)	30.8% (7.9 to 57)		4498	618.5 (96 to 1067)	15.9% (2.2 to 31.1)
Pneumococcal conjugate (2)	1141	-162 (-424.6 to 43)	-12.4% (-27.1 to 3.9)		1530	418 (192 to 606)	37.6% (14.3 to 65.6)		1653	338 (50.4 to 597.3)	25.7% (3.1 to 56.6)		4324	584 (-14.6 to 1146.6)	15.6% (-0.3 to 36.1)
Pneumococcal conjugate (3)	1041	-298.5 (-589 to -103)	-22.3% (-36.1 to -9)		1541	328 (78.8 to 549)	27% (5.4 to 55.3)		1611	251.5 (-49.5 to 468.5)	18.5% (-3 to 41)		4193	279.5 (-362.8 to 793.1)	7.1% (-8 to 23.3)
Rotavirus (1)	1116	-619 (-908 to -419.3)	-35.7% (-44.9 to -27.3)		1607	79 (-233.2 to 246.6)	5.2% (-12.7 to 18.1)		1772	-215.5 (-713.6 to 23.5)	-10.8% (-28.7 to 1.3)		4495	-761.5 (-1602.4 to -282.2)	-14.5% (-26.3 to -5.9)
Rotavirus (2)	961	-618 (-859.2 to -457)	-39.1% (-47.2 to -32.2)		1280	-183.5 (-476 to -33)	-12.5% (-27.1 to -2.5)		1653	-223.5 (-632.7 to -34.1)	-11.9% (-27.7 to -2)		3894	-1041.5 (-1667.5 to -656.9)	-21.1% (-30 to -14.4)
Measles (1)	915	-422 (-729.5 to -158.9)	-31.6% (-44.4 to -14.8)		1552	373 (78.4 to 617.5)	31.6% (5.3 to 66.1)		1488	70 (-344.2 to 394.6)	4.9% (-18.8 to 36.1)		3955	15 (-810 to 716.5)	0.4% (-17 to 22.1)
**Malawi**															
Bacillus Calmette–Guerin (1)	3110	101 (-322.7 to 392.5)	3.4% (-9.4 to 14.4)		3046	-470.5 (-887.2 to -181)	-13.4% (-22.6 to -5.6)		2751	-596 (-1146.8 to -218.2)	-17.8% (-29.4 to -7.3)		8907	-977.5 (-2051.9 to -219)	-9.9% (-18.7 to -2.4)
Oral or inactivated polio (0)	NA	NA	NA		NA	NA	NA		NA	NA	NA		NA	NA	NA
Oral or inactivated polio (1)	5969	139 (-373 to 529.4)	2.4% (-5.9 to 9.7)		5893	-1296.5 (-1936.7 to -855.9)	-18% (-24.7 to -12.7)		6157	-1749 (-2537.1 to -1180.5)	-22.1% (-29.2 to -16.1)		18019	-2958.5 (-4320.1 to -1823)	-14.1% (-19.3 to -9.2)
Oral or inactivated polio (2)	2898	396.5 (214 to 568.5)	15.9% (8 to 24.4)		2799	-133.5 (-364.7 to 63.5)	-4.6% (-11.5 to 2.3)		2674	-74 (-304.2 to 137)	-2.7% (-10.2 to 5.4)		8371	181 (-303 to 639.3)	2.2% (-3.5 to 8.3)
Oral or inactivated polio (3)	3096	370 (134.5 to 544.5)	13.6% (4.5 to 21.3)		2993	-296.5 (-532.1 to -94.4)	-9% (-15.1 to -3.1)		2655	-439.5 (-728.1 to -228.4)	-14.2% (-21.5 to -7.9)		8744	-364.5 (-980.8 to 99.5)	-4% (-10.1 to 1.2)
Pentavalent (1)	2976	269 (69.9 to 434)	9.9% (2.4 to 17.1)		2861	-409.5 (-627.6 to -214)	-12.5% (-18 to -7)		2692	-301 (-547.1 to -99.1)	-10.1% (-16.9 to -3.6)		8529	-453 (-964.2 to -36)	-5% (-10.2 to -0.4)
Pentavalent (2)	2904	399 (234.9 to 567.5)	15.9% (8.8 to 24.3)		2683	-248 (-474.6 to -42)	-8.5% (-15 to -1.5)		2494	-247.5 (-472.5 to -23)	-9% (-15.9 to -0.9)		8081	-103.5 (-525.1 to 346.6)	-1.3% (-6.1 to 4.5)
Pentavalent (3)	3119	398.5 (179.8 to 573)	14.6% (6.1 to 22.5)		3001	-236 (-470 to -22.9)	-7.3% (-13.5 to -0.8)		2793	-261 (-512.1 to -23.5)	-8.5% (-15.5 to -0.8)		8913	-105 (-714.3 to 397.6)	-1.2% (-7.4 to 4.7)
Pneumococcal conjugate (1)	2952	278 (83.5 to 442.1)	10.4% (2.9 to 17.6)		2830	-409 (-631.6 to -219.8)	-12.6% (-18.2 to -7.2)		2788	-168.5 (-420.4 to 51)	-5.7% (-13.1 to 1.9)		8570	-302.5 (-845.1 to 184.8)	-3.4% (-9 to 2.2)
Pneumococcal conjugate (2)	2889	384 (197.5 to 558)	15.3% (7.3 to 23.9)		2749	-184 (-436.7 to 3.7)	-6.3% (-13.7 to 0.1)		2641	-115.5 (-362.1 to 87.6)	-4.2% (-12.1 to 3.4)		8279	81.5 (-454.1 to 540.1)	1% (-5.2 to 7)
Pneumococcal conjugate (3)	3096	374 (183.9 to 535)	13.7% (6.3 to 20.9)		2978	-287 (-486.6 to -95.3)	-8.8% (-14 to -3.1)		2792	-257 (-481.1 to -59.4)	-8.4% (-14.7 to -2.1)		8866	-173.5 (-635.5 to 278.5)	-1.9% (-6.7 to 3.2)
Rotavirus (1)	2946	234 (43 to 386)	8.6% (1.5 to 15.1)		2934	-507.5 (-738.1 to -308)	-14.7% (-20.1 to -9.5)		2840	-234 (-463.5 to -27.4)	-7.6% (-14 to -1)		8720	-520 (-992.6 to -48.9)	-5.6% (-10.2 to -0.6)
Rotavirus (2)	3039	465.5 (296.4 to 623)	18.1% (10.8 to 25.8)		2778	-302 (-530 to -124)	-9.8% (-16 to -4.3)		2570	-317.5 (-576.1 to -110.5)	-11% (-18.3 to -4.1)		8387	-174 (-656.6 to 292)	-2% (-7.3 to 3.6)
Measles (1)	2868	722 (168.5 to 1019)	33.6% (6.2 to 55.1)		2888	-118 (-700.6 to 321)	-3.9% (-19.5 to 12.5)		2517	366 (-176.1 to 658)	17% (-6.5 to 35.4)		8273	926 (-232.9 to 1710.6)	12.6% (-2.7 to 26.1)

NA: data not available; PI: prediction interval.

Note: Cumulative differences across three periods will only approximately sum to cumulative difference for entire period as we report the median value across bootstrap samples.

### Vaccinations, September 2020–February 2021

Early in the middle evaluation period (September–November 2020), most of the vaccinations administered early in childhood returned to within expected levels in all four countries (Fig. 1, Fig. 2, Fig. 3, Fig. 4 and Fig. 5). However, in the second half of this evaluation period, the expected vaccination numbers for dose 3 vaccines and the measles vaccine decreased in Lesotho, BCG vaccination decreased in Haiti, and BCG and doses 1–3 vaccination remained at expected or decreased levels in Malawi (Fig. 1, Fig. 2, Fig. 3, Fig. 4 and Fig. 5). Liberia maintained vaccinations at predicted levels throughout the period except for sharp decreases in pentavalent vaccinations. For September 2020–February 2021, the median (range) cumulative percentage difference in the 14 vaccine-dose combinations reported was 6.9% (−37.5% to 17.6%) greater than expected for Haiti, 11.5% (−15.7% to 17.4%) less for Lesotho, 27.8% (−12.5% to 56.5%) greater for Liberia and 9.0% (−18.0% to −3.9%) less for Malawi (Table 4); details in data repository.^28^

### Vaccinations, March–August 2021

In March–April 2021, most of the vaccinations returned to almost expected levels or above in Malawi; Haiti, Lesotho and Liberia maintained vaccination levels (Fig. 1, Fig. 2, Fig. 3, Fig. 4 and Fig. 5). However, from May to August 2021, all vaccinations in Malawi decreased from expected levels and in Liberia polio and rotavirus vaccinations decreased (Fig. 1, Fig. 2, Fig. 3, Fig. 4 and Fig. 5). For March–August 2021 the median (range) cumulative percentage difference in the 14 vaccine-dose combinations reported was 2.1% (−21.5% to 43.2%) greater than expected for Haiti, 5.7% (−16.9% to 16.1%) greater for Lesotho, 9.6% (−20.8% to 32.0 %) greater for Liberia and 8.5% (−22.1% to 17%) less for Malawi (Table 4); details in data repository.^28^

### Overall vaccination deficits

For the whole evaluation period (March 2020–August 2021), all countries except Liberia had a cumulative deficit. The median (range) cumulative percentage difference in the 14 vaccine-dose combinations reported was 5.1% (−16.8% to 19.9%) less than expected for Haiti, 5.9% (−16.3% to 10.1%) less for Lesotho, 8.0% (−21.1% to 15.9%) greater for Liberia and 2.0% (−14.1% to 12.6%) less for Malawi (Table 4); details in data repository.^28^ The deficit was significant for only a few vaccine-dose combinations: rotavirus doses 1 and 2 for Liberia, and all vaccine-dose combinations in Malawi.

## Discussion

Pandemics can disrupt infrastructure and divert health resources. From four low- and middle-income countries in sub-Saharan Africa and the Caribbean, we found that vaccine service utilization was affected for children younger than 1 year, largely during the early months of the COVID-19 pandemic and during subsequent waves of infection. Early declines in vaccinations, with decreases of up to 75%, were also observed in the Netherlands,^19^ Pakistan,^17^ Singapore,^21^ United Kingdom of Great Britain and Northern Ireland^18^ and the United States of America (USA)^20^ during full physical-distancing measures or restriction periods in early 2020. However, and importantly, the initial declines were not sustained in the facilities we studied, with rebounds to expected levels in all vaccines observed within 3 months of the initial pandemic period and in between waves of infection. These results are similar to the findings of two recently published studies on vaccination uptake. Across eight African countries from March to July 2020, initial declines were seen in immunization with the pentavalent 3 and BCG vaccines but the levels had returned to normal by July 2020.^32^ In another study on health-service utilization in Kinshasa, Democratic Republic of the Congo until December 2020, no overall decrease in vaccine uptake was seen for children aged 12 months or younger.^22^ We found that similar trends persisted in our study locations well into 2021.

In our study, all countries except Malawi showed early declines in vaccination uptake, with Lesotho having the smallest disruptions and Liberia and Haiti having the largest near equal disruptions. The reasons for variations in vaccine coverage are likely complex and varied by country, and even between districts within a country. For example, initial restrictions during the COVID-19 pandemic differed between countries with Haiti, Liberia and Lesotho having very high initial COVID-19 stringency indexes in April 2020.^29^ However, often the restrictions were not strictly adhered to in rural districts as seen across rural Africa,^33^ which may account for the maintenance in immunization in Malawi. Furthermore, transient declines in BCG and polio vaccinations in Lesotho in October 2020 were due to stock-outs, demonstrating the pandemic’s effect on supply chains.^12^^,^^34^ Lastly, individuals were likely hesitant to visit health-care facilities, either because of the perceived risk of being infected by SARS-CoV-2 or problems with travel during restrictions. Reduced health-seeking behaviour for immunizations has been observed in past pandemics and outbreak emergencies^6^^,^^7^ and studies early in the COVID-19 pandemic have suggested similar results.^17^^–^^21^^,^^32^

The later declines in vaccination in 2021 corresponded with peaks in infection with higher cumulative cases and case fatality rates (Table 1). Malawi and Lesotho had large waves of infection in January–March 2021 and May–August 2021, which matched declines in several vaccinations. These declines were most pronounced in Malawi, probably because of a lack of community outreach during infection waves (Table 1). The declines in immunization are most likely due to renewed fear of visiting health-care facilities coupled with increased restrictive measures.^33^^,^^35^ In addition, stock-outs of vaccines in Haiti and Liberia contributed to discrete drops in administration of BCG and pentavalent vaccines (Table 1).^34^

Generally, we found that the declines were least pronounced in vaccines administered at the time of birth (BCG vaccine and polio vaccine dose 0), except for transient stock-outs in Lesotho and Haiti and large infection waves in Lesotho and Malawi. The declines became more pronounced as the recommended age for a child to receive the vaccine increased. All sites had statistically significant declines in measles vaccinations administered at 9 months, especially early in the pandemic and with waves of infection, a concern that has been flagged for several low- and middle-income countries.^36^ Even with restrictive measures and fear of infection, delivery in a health facility was seen as a vital health service during the pandemic,^37^ which explains the small decline in the uptake of vaccines administered immediately after birth. Later vaccines often required families to travel from home to facilities; parental fear of exposing their children to infection during vaccination is a key challenge that has been observed in other studies.^16^^,^^17^^,^^33^^,^^38^

Few studies thus far have reported trends in vaccinations past the early COVID-19 pandemic period in 2020. In the 10 districts in our study, uptake of childhood vaccines for infants aged 12 months or younger returned to or were maintained at expected levels within 6 months of the start of the COVID-19 pandemic and with periods of low COVID-19 infection rates. This finding is consistent with the one other study we identified that had extended monitoring, where the administration of first measles, mumps and rubella vaccine in the Netherlands returned to within 1–2% of baseline by September 2020.^19^ The eight African countries study, which only followed immunizations until July 2020, observed a return to normal levels within 4 months.^32^ Even with the returns to expected levels in monthly administration throughout the pandemic, all countries in our study, except Liberia, had an overall cumulative decrease in median vaccination administration of between 2% and 6%. However, the only significant deviations were in both rotavirus doses in Liberia and all vaccinations in Malawi.

The maintenance of or return to close-to-normal monthly administration in Liberia, Haiti and Lesotho also likely has several reasons, including lifting of the strict restrictive measures, fewer COVID-19 cases, better public understanding of the pandemic and infection risks, and community outreach to improve vaccination.^18^^–^^20^ In addition, the Partners In Health and district-level health ministry teams adopted several strategies to support vaccination administration during the pandemic. For example, our sites supported information campaigns through various outlets consistent with WHO recommendations for robust community health education^39^ and clinical and preventive services for promotion of essential services. Other studies have shown that public health messages to encourage essential services such as vaccinations by national and international governing bodies^39^ and community and/or individual vaccination awareness campaigns^16^^,^^19^ have supported the uptake of vaccination services during the COVID-19 pandemic. In addition, providing extra staffing and space to separate children visiting clinics for immunizations was critical for maintaining vaccination uptake in the USA during the pandemic.^40^

Furthermore, our teams provided logistical support for routine immunization campaigns, social or transport support and Liberia and Lesotho reported supporting logistics and incentives in special community catch-up immunization campaigns in their districts, which have been used elsewhere to maintain and increase vaccination coverage.^11^^,^^16^^,^^17^^,^^19^^,^^41^ Despite this support, it is important to note that some teams reported persistent challenges in staffing, availability of safe spaces for vaccination, transport and cold chain equipment for separate or outreach vaccination; for example, in Malawi, especially during the second and third waves of infection, community outreach was suspended. Lastly, the COVID-19 vaccine campaign was introduced in all sites in March–May 2021. This factor may have exacerbated COVID-19 vaccine misinformation campaigns^42^ and decreased utilization of routine immunization but we did not directly study this effect. Further work to understand the influence of the introduction of COVID-19 vaccination on routine immunization is important. These issues must be addressed to maintain coverage during new waves of the COVID-19 pandemic and to reach and immunize the children missed during the early months of the pandemic.

Our study has several limitations. First, the data used from the District Health Information Software 2 are aggregated at the facility level. We cannot assess whether individual patients received all vaccines, if vaccinations were on time or delayed for a specific child, or if the catchment populations of the districts changed from the baseline period. Another challenge is the possibility that the COVID-19 pandemic affected the timeliness and completeness of facility-level reporting, particularly during restrictions; however, Partners In Health teams provided additional logistical and technical resources to ensure continued timely data collection in these 10 districts. Furthermore, the data are limited to the 10 districts within the four countries; while this may limit generalizability, these results represent a broad geography not yet included in reports on the effect of the COVID-19 pandemic.

Another important limitation is the exclusion of facilities with more than 20% missing baseline data. Country teams raised concerns that high levels of missing data at a facility could also suggest poor accuracy of the data reported, which in turn could lead to inaccurate models and predicted counts during the overall period. For this reason, in our ongoing monitoring, we do not report on sites with high levels of missing data. The country-level measures reported in our study are aggregated across the facility-level model predictions and observed values;^25^ therefore, facilities excluded from the ongoing monitoring are excluded from these aggregate measures. While this may lead to an undercount in both the predicted and observed number of immunizations, we believe that the per cent deviations and significance of deviations are more accurate by excluding facilities with high levels of missing data. Our teams are currently exploring imputation methods so that facilities with high levels of missing data can still be included in these monitoring activities.^43^ However, it is important to emphasize that we excluded very few facilities because of having more than 20% missing data, with a maximum number of four facilities, out of 41 facilities, excluded for a single vaccine dose indicator.

To ensure vaccine utilization rates are maintained despite continued waves of infection and potential renewed restrictions, we suggest governments and health-care stakeholders strengthen efforts for educating communities and parents on COVID-19 risks and the value of childhood vaccinations with targeted community outreach clinics.

## References

[1] Hartley DM, Perencevich EN. Public health interventions for COVID-19: emerging evidence and implications for an evolving public health crisis. JAMA. 2020 May 19;323(19):1908–9. 10.1001/jama.2020.5910 32275299

[2] Horton S. Cost-effectiveness analysis in disease control priorities. In: Jamison DT, Gelband H, Horton S, Jha P, Mock CN, Nugent R, editors. Disease control priorities: improving health and reducing poverty. Washington, DC: The International Bank for Reconstruction and Development/The World Bank; 2017. 10.1596/978-1-4648-0527-1_ch7 10.1596/978-1-4648-0527-1_ch7 30212058

[3] Lee LA, Franzel L, Atwell J, Datta SD, Friberg IK, Goldie SJ, et al. The estimated mortality impact of vaccinations forecast to be administered during 2011-2020 in 73 countries supported by the GAVI Alliance. Vaccine. 2013 Apr 18;31 Suppl 2:B61–72. 10.1016/j.vaccine.2012.11.035 23598494

[4] Ozawa S, Mirelman A, Stack ML, Walker DG, Levine OS. Cost-effectiveness and economic benefits of vaccines in low- and middle-income countries: a systematic review. Vaccine. 2012 Dec 17;31(1):96–108. 10.1016/j.vaccine.2012.10.103 23142307

[5] State of inequality: childhood immunization. Geneva: World Health Organization; 2016. Available from: https://apps.who.int/iris/handle/10665/252541 [cited 2021 Mar 11].

[6] Lau JT, Yang X, Pang E, Tsui HY, Wong E, Wing YK. SARS-related perceptions in Hong Kong. Emerg Infect Dis. 2005 Mar;11(3):417–24. 15757557 10.3201/eid1103.040675 PMC3298267

[7] Truelove SA, Moss WJ, Lessler J. Mitigating measles outbreaks in West Africa post-Ebola. Expert Rev Anti Infect Ther. 2015;13(11):1299–301. 10.1586/14787210.2015.1085305 26489536

[8] Parpia AS, Ndeffo-Mbah ML, Wenzel NS, Galvani AP. Effects of response to 2014–2015 Ebola outbreak on deaths from malaria, HIV/AIDS, and tuberculosis, West Africa. Emerg Infect Dis. 2016 Mar;22(3):433–41. 10.3201/eid2203.150977 26886846 PMC4766886

[9] Spencer N, Nathawad R, Arpin E, Johnson S. Pandemics, epidemics and inequities in routine childhood vaccination coverage: a rapid review. BMJ Paediatr Open. 2020 11 2;4(1):e000842. 10.1136/bmjpo-2020-000842 33195821 PMC7607602

[10] Abbas K, Procter SR, van Zandvoort K, Clark A, Funk S, Mengistu T, et al. Routine childhood immunisation during the COVID-19 pandemic in Africa: a benefit-risk analysis of health benefits of routine childhood immunisation against the excess risk of SARS-CoV-2 infections during the COVID-19 pandemic in Africa. Lancet Glob Health. 2020 Oct;8(10):e1264–72. 10.1016/S2214-109X(20)30308-9 32687792 PMC7367673

[11] Nelson R. COVID-19 disrupts vaccine delivery. Lancet Infect Dis. 2020 May;20(5):546. 10.1016/S1473-3099(20)30304-2 32311326 PMC7164887

[12] Roberton T, Carter ED, Chou VB, Stegmuller AR, Jackson BD, Tam Y, et al. Early estimates of the indirect effects of the COVID-19 pandemic on maternal and child mortality in low-income and middle-income countries: a modelling study. Lancet Glob Health. 2020 Jul;8(7):e901–8. 10.1016/S2214-109X(20)30229-1 32405459 PMC7217645

[13] Wong CA, Ming D, Maslow G, Gifford EJ. Mitigating the impacts of the COVID-19 pandemic response on at-risk children. Pediatrics. 2020 Jul;146(1):e20200973. 10.1542/peds.2020-0973 32317311 PMC8610088

[14] Haider N, Osman AY, Gadzekpo A, Akipede GO, Asogun D, Ansumana R, et al. Lockdown measures in response to COVID-19 in nine sub-Saharan African countries. BMJ Glob Health. 2020 Oct;5(10):e003319. 10.1136/bmjgh-2020-003319 33028699 PMC7542624

[15] Pulse survey on continuity of essential health services during the COVID-19 pandemic: interim report, 27 August 2020. Geneva: World Health Organization; 2020. Available from: https://apps.who.int/iris/handle/10665/334048 [cited 2021 Mar 11].

[16] Alsuhaibani M, Alaqeel A. Impact of the COVID-19 pandemic on routine childhood immunization in Saudi Arabia. Vaccines (Basel). 2020 Oct 3;8(4):581. 10.3390/vaccines8040581 33022916 PMC7711657

[17] Chandir S, Siddiqi DA, Setayesh H, Khan AJ. Impact of COVID-19 lockdown on routine immunisation in Karachi, Pakistan. Lancet Glob Health. 2020 Sep;8(9):e1118–20. 10.1016/S2214-109X(20)30290-4 32615076 PMC7324087

[18] McDonald HI, Tessier E, White JM, Woodruff M, Knowles C, Bates C, et al. Early impact of the coronavirus disease (COVID-19) pandemic and physical distancing measures on routine childhood vaccinations in England, January to April 2020. Euro Surveill. 2020 May;25(19):2000848. 10.2807/1560-7917.ES.2020.25.19.2000848 32431288 PMC7238742

[19] Middeldorp M, van Lier A, van der Maas N, Veldhuijzen I, Freudenburg W, van Sorge NM, et al. Short term impact of the COVID-19 pandemic on incidence of vaccine preventable diseases and participation in routine infant vaccinations in the Netherlands in the period March–September 2020. Vaccine. 2021 Feb 12;39(7):1039–43. 10.1016/j.vaccine.2020.12.080 33478793 PMC7787078

[20] O’Leary ST, Trefren L, Roth H, Moss A, Severson R, Kempe A. Number of childhood and adolescent vaccinations administered before and after the COVID-19 outbreak in Colorado. JAMA Pediatr. 2021 Mar 1;175(3):305–7. 10.1001/jamapediatrics.2020.4733 33284331 PMC7921904

[21] Zhong Y, Clapham HE, Aishworiya R, Chua YX, Mathews J, Ong M, et al. Childhood vaccinations: hidden impact of COVID-19 on children in Singapore. Vaccine. 2021 Jan 29;39(5):780–5. 10.1016/j.vaccine.2020.12.054 33414050 PMC7762701

[22] Hategeka C, Carter SE, Chenge FM, Katanga EN, Lurton G, Mayaka SM-N, et al. Impact of the COVID-19 pandemic and response on the utilisation of health services in public facilities during the first wave in Kinshasa, the Democratic Republic of the Congo. BMJ Glob Health. 2021 Jul;6(7):e005955. 10.1136/bmjgh-2021-005955 34315776 PMC8318723

[23] Spencer N, Nathawad R, Arpin E, Johnson S. Pandemics, epidemics and inequities in routine childhood vaccination coverage: a rapid review. BMJ Paediatr Open. 2020 Nov 2;4(1):e000842. 10.1136/bmjpo-2020-000842 33195821 PMC7607602

[24] Partners In Health [internet]. Boston: Partners In Health; 2021. Available from: www.pih.org [cited 2021 May 25].

[25] Fulcher IR, Boley EJ, Gopaluni A, Varney PF, Barnhart DA, Kulikowski N, et al.; Cross-site COVID-19 Syndromic Surveillance Working Group. Syndromic surveillance using monthly aggregate health systems information data: methods with application to COVID-19 in Liberia. Int J Epidemiol. 2021 Aug 30;50(4):1091–102. 10.1093/ije/dyab094 34058004 PMC8195038

[26] Dhis2 in action [internet]. Oslo: Dhis2; 2021. Available from: https://www.dhis2.org/in-action [cited 2020 Dec 1].

[27] WHO toolkit for routine health information systems data. Geneva: World Health Organization; 2021. Available from: https://www.who.int/data/data-collection-tools/health-service-data/toolkit-for-routine-health-information-system-data/modules [cited 2021 Nov 7].

[28] Connolly E, Boley J, Fejfar DL, Varney PF, Aron MB, Fulcher IR, et al. Childhood immunization during the COVID-19 pandemic: experiences in Haiti, Lesotho, Liberia and Malawi (supplementary material). Geneva: Zenodo; 2021. 10.5281/zenodo.5675482 PMC8795848 35125536

[29] Hale T, Angrist N, Goldszmidt R, Kira B, Petherick A, Phillips T, et al. A global panel database of pandemic policies (Oxford COVID-19 Government Response Tracker). Nat Hum Behav. 2021 Apr;5(4):529–38. 10.1038/s41562-021-01079-8 33686204

[30] Dong E, Du H, Gardner L. An interactive web-based dashboard to track COVID-19 in real time. Lancet Infect Dis. 2020 05;20(5):533–4. 10.1016/S1473-3099(20)30120-1 32087114 PMC7159018

[31] Case fatality rate of COVID-19 [internet]. Oxford: Our World in Data; 2021. Available from: https://ourworldindata.org/explorers/coronavirus-data-explorer?zoomToSelection=true&time=2020-03-14.latest&facet=none&pickerSort=asc&pickerMetric=location&hideControls=true&Metric=Case+fatality+rate&Interval=Cumulative&Relative+to+Population=false&Align+outbreaks=true&country=MWI~LBR~LSO~HTI [cited 2021 Oct 23].

[32] Shapira G, Ahmed T, Drouard SHP, Amor Fernandez P, Kandpal E, Nzelu C, et al. Disruptions in maternal and child health service utilization during COVID-19: analysis from eight sub-Saharan African countries. Health Policy Plan. 2021 Aug 12;36(7):1140–51. 10.1093/heapol/czab064 34146394 PMC8344431

[33] Okereke M, Ukor NA, Ngaruiya LM, Mwansa C, Alhaj SM, Ogunkola IO, et al. COVID-19 misinformation and infodemic in rural Africa. Am J Trop Med Hyg. 2021;104(2):453–6. 10.4269/ajtmh.20-1488 33382028 PMC7866344

[34] At least 80 million children under one at risk of diseases such as diphtheria, measles and polio as COVID-19 disrupts routine vaccination efforts warn GAVI, WHO and UNICEF [internet]. Geneva: World Health Organization; 2020. Available from: https://www.who.int/news/item/22-05-2020-at-least-80-million-children-under-one-at-risk-of-diseases-such-as-diphtheria-measles-and-polio-as-covid-19-disrupts-routine-vaccination-efforts-warn-gavi-who-and-unicef [cited 2021 Nov 7].

[35] Haider N, Osman AY, Gadzekpo A, Akipede GO, Asogun D, Ansumana R, et al. Lockdown measures in response to COVID-19 in nine sub-Saharan African countries. BMJ Glob Health. 2020 Oct;5(10):e003319. 10.1136/bmjgh-2020-003319 33028699 PMC7542624

[36] Bagcchi S. Measles immunisation gaps in Africa. Lancet Infect Dis. 2021 Jul;21(7):918. 10.1016/S1473-3099(21)00340-6 34174232 PMC8443021

[37] Ahmed T, Rahman AE, Amole TG, Galadanci H, Matjila M, Soma-Pillay P, et al. The effect of COVID-19 on maternal newborn and child health (MNCH) services in Bangladesh, Nigeria and South Africa: call for a contextualised pandemic response in LMICs. Int J Equity Health. 2021 Mar 15;20(1):77. 10.1186/s12939-021-01414-5 33722225 PMC7957460

[38] Hoffman J. Vaccination rates drop dangerously as parents avoid doctor’s visits. The New York Times. 2020 Apr 23. Available from: https://www.nytimes.com/2020/04/23/health/coronavirus-measles-vaccines.html [cited 2020 Apr 23].

[39] Immunization as an essential health service: guiding principles for immunization activities during the COVID-19 pandemic and other times of severe disruption, 1 November 2020. Geneva: World Health Organization; 2020. Available from: https://apps.who.int/iris/handle/10665/336542 [cited 2021 May 23].

[40] Santoli JM, Lindley MC, DeSilva MB, Kharbanda EO, Daley MF, Galloway L, et al. Effects of the COVID-19 pandemic on routine pediatric vaccine ordering and administration – United States, 2020. MMWR Morb Mortal Wkly Rep. 2020 May 15;69(19):591–3. 10.15585/mmwr.mm6919e2 32407298

[41] Isaac MR, Chartier M, Brownell M, Chateau D, Nickel NC, Martens P, et al.; PATHS Equity Team Members. Can opportunities be enhanced for vaccinating children in home visiting programs? A population-based cohort study. BMC Public Health. 2015 Jul 7;15(1):620. 10.1186/s12889-015-1926-8 26149681 PMC4494701

[42] COVID-19 putting routine childhood immunization in danger: UN health agency. UN News. 2020 Apr 27. Available from: https://news.un.org/en/story/2020/04/1062712 [cited 2021 Mar 21].

[43] Feng S, Hategeka C, Grépin KA. Addressing missing values in routine health information system data: an evaluation of imputation methods using data from the Democratic Republic of the Congo during the COVID-19 pandemic. Popul Health Metr. 2021 11 4;19(1):44. 10.1186/s12963-021-00274-z 34736462 PMC8567342

